# Supplementary material for: A set of multi-entry identification keys to African frugivorous flies (Diptera, Tephritidae)
Source: Zookeys. 2014 Jul 24;(428):97–108. doi: 10.3897/zookeys.428.7366 (PMC4143993; doi:10.3897/zookeys.428.7366)
Supplement: Supplementary material 6 — Key to Ceratitis [file zookeys-428-097-s006.zip › SF6_ZooKeys_key to Ceratitis/key/SF6_ZooKeys_key to Ceratitis/Media/Html/Ceratitis quinaria .htm]

Ceratitis (Ceratalaspis) quinaria (Bezzi)


***Ceratitis*** **(*Ceratalaspis*)
*quinaria* (Bezzi)**

*Pardalaspis
quinaria*
Bezzi, 1918: 235.

Body
length: 4.20 (3.60-4.75) mm; wing length: 3.93 (3.50-4.50) mm.

Male

Head.
Antenna yellow to yellow-orange. Third antennal segment twice as long as second
segment. Arista with short hairs over entire length. Frons pale yellow, centre
usually deeper yellow coloured; with short scattered hairs which are largely
the same colour as frons; concave to flat, in lateral view not distinctly
projecting forwards at antennal implant. Chaetotaxy normal for subgenus, except
more reddish.

Thorax.
Postpronotum white or white-yellow, unspotted. Ground colour of mesonotum pale;
mesonotal pattern variable, dorsocentral spots often less distinct. Chaetotaxy
normal for subgenus. Scapular setae pale. One anepisternal bristle. Scutellum
white basally, otherwise yellow with three apical and two subapical small black
spots; basally without dark spots, only slight darker colouration. Subscutellum
completely pale.

Legs
yellow; setation typical for subgenus, mainly pale especially on femora.
Posterior and posterodorsal rows on fore femur pale.

Wing
bands with colour of markings variable, yellow to yellow-brown. Banding,
marginal band continuous; cubital band free; medial band absent; crossvein r-m
well before middle of discal cell, usually at basal third. Crossvein dm-cu
position variable.

Abdomen.
Pale yellow colour. Setation and banding typical for subgenus.

Female

As
male except for the following characters: Scutellum in Aden specimen with
lateral sets of spots partly fused. Oviscape shorter than abdominal terga 3-6
combined.

(Description
after De Meyer, 1998)

�
